# How children with posttraumatic stress disorder view their social networks: a qualitative study

**DOI:** 10.1007/s40653-026-00896-1

**Published:** 2026-04-29

**Authors:** Mèlanie Sloover, Dani De Beijer, Sabine E. M. J. Stoltz, Roxanna Camfferman, Ramón J. L. Lindauer, Elisa Van Ee

**Affiliations:** 1 Radboud University, Behavioural Science Institute, Nijmegen, Netherlands; 2 Reinier van Arkel, Psychotraumacentrum Zuid Nederland, s-Hertogenbosch, Netherlands; 3 Reinier van Arkel, Herlaarhof, Vught, Netherlands; 4 Academic Center for Child and Adolescent Psychiatry, Levvel, Amsterdam, Netherlands; 5 Department of Child and Adolescent Psychiatry, Amsterdam University Medical Centers, Amsterdam, Netherlands

**Keywords:** Post-traumatic stress disorder, Social network, Childhood trauma, Interpersonal trauma

## Abstract

Research on the social networks of children with posttraumatic stress disorder (PTSD) remains limited. Although a link between PTSD symptoms and the child’s social environment is often assumed, this can be impacted in cases of interpersonal trauma. This is important, because social support can play a beneficial role in recovery after experiencing trauma. However, little is known about how children with interpersonal trauma perceive their social network and the support they gain from this network. This qualitative study used semi-structured interviews to explore these perceptions in greater depth. Ten children who experienced interpersonal trauma between the ages of 8 to 12 were interviewed about their social network. The content of the interviews was analyzed using reflexive thematic analysis. Descriptive statistics showed that children had varied networks with 40% of relationships experienced as ambivalent or negative. In addition, there was a general tendency to not discuss their traumatic experiences. Thematic analysis identified four key themes. First of all, the parent-child relationship was often strained with both parents, but in different ways. Second, there seemed to be a contrast between the positive definition children gave of friendship and the negative experiences they had within their own reported friendships. Third, children were able to identify supportive actions from different individuals in their network, related to the trauma as well as more general support. Finally, children reported various reasons for their general reluctance to discuss their trauma. The findings emphasize a need for help with strengthening the children’s social network to improve recovery from trauma.

Being exposed to adversity in childhood such as sexual abuse, violence, (traffic) accidents, domestic violence, or natural disasters has been associated with mental health problems, in particular post-traumatic stress disorder (PTSD; McLaughlin et al., 2010). A recent meta-analysis revealed that 12% of children (ages 1–18) met the criteria for a PTSD classification based on the DSM-V after exposure to childhood adversity (Visser et al., 2025). Children and adolescents exposed to interpersonal trauma such as sexual or domestic violence were most at risk, possibly due to these events often being more chronic or leading to more self-blame than non-interpersonal events (Alisic et al., 2014). This type of chronic interpersonal trauma in the child-rearing context is commonly referred to as developmental trauma and is often paired with attachment disruptions (Ford, 2023). Developmental trauma can have wide-ranging consequences beyond PTSD, such as impaired cognitive functioning, behavioral dysregulation, and affected self-concept (Van der Kolk, 2005). Research has shown that parental reactions after child trauma exposure and peer support impact the development of PTSD in children (Dyregrov & Yule, 2006; Morley & Kohrt, 2014; Xiong et al., 2022). These studies show that lower levels of social support from parents and peers and parents avoiding discussion of the events are associated with higher levels of PTSD in children. However, these studies mainly included samples with single event trauma exposure. So, while social support may serve a protective role in children exposed to childhood adversity, it remains unclear whether similar protective processes operate in the context of developmental trauma, which often occurs within caregiving relationships and may affect children’s social networks, potentially limiting the availability or effectiveness of such support.

Receiving social support is conditional on having a social network — that is, a set of meaningful social relationships capable of providing such support. Social support entails the perceived availability of and satisfaction with support, as well as actual actions taken by another individual to help (Barrera, 1986). Indeed, previous meta-analyses have revealed that perceived as well as enacted social support (or a lack thereof) is one of the most important predictors of the development of PTSD in adults (Wang et al., 2021; Brewin et al., 2000; Zalta et al., 2021). In addition, research in adults with PTSD finds that positive interactions with an individual’s social network can facilitate recovery from PTSD, while negative interactions contribute to the persistence of PTSD symptoms (Charuvastra & Cloitre, 2008). One important factor that makes a social network especially protective against PTSD is diversity. A study showed that adults with a more diverse social network experience greater benefits than those whose social networks are less diverse (Platt et al., 2014). Diversity was operationalized as the number of different social roles someone has in their network. This diversity may relate to an increase in perceived social support, which in turn relates to the added benefit. In sum, it is clear that social support and specific characteristics of the social network can play a beneficial role in PTSD. However, the previously cited literature is based on adults. It is less clear how the social network and the support it provides plays a role in children with PTSD, while trauma exposure and PTSD are also common among children.

For children, their social network generally consists of family, peers, and teachers (Lewis, 2005). Support from these groups is known to be positively related to general mental well-being of children and adolescents (e.g. Butler et al., 2022; Chu et al., 2010; Bokhorst et al., 2010). A recent meta-analysis looked into the protective role of social support specifically for PTSD in children. The results show that peer, family, and teacher support are related to severity of PTSD symptoms, with higher support relating to lower symptom severity (Allen et al., 2021). Importantly, the included studies mainly regarded adolescents who had experienced a single-event trauma such as a natural disaster and the effect sizes were small. In addition, research in adults has shown that PTSD symptoms can negatively impact social support (Laffaye et al., 2008; Wang et al., 2021). The studies in the recent meta-analysis also show mixed findings across different age groups, with limited research on younger children, especially in the context of developmental trauma. However, the role of the social network changes over time with childhood friendships often being more unstable than adolescent or adult ones and higher dependence on parent-child relationships (Jackson-Dwyer, 2014). At the same time, children who have experienced interpersonal trauma in the family context may be unable to depend on their parents. Therefore, more research is needed to determine how social support within the network of younger children who have experienced interpersonal trauma relates to PTSD symptoms.

Looking at specific sources of support in greater detail might shed light on when support is perceived as effective. One review concluded that parents communicating with their children about potentially traumatic experiences in a supportive (sensitive and responsive) way seems to be beneficial for children’s well-being (Sloover et al., 2024). In addition, a study on adolescents who had experienced sexual abuse and reported clinical levels of PTSD symptoms revealed that both support from peers and mothers were protective factors against PTSD severity (Hébert et al., 2014). However, the studies in that review mainly included children who had experienced a single traumatic event, while interpersonal trauma in the family context is often more chronic in nature. From an attachment perspective (Bowlby, 1988), chronic interpersonal trauma within the caregiving context may disrupt the child’s sense of safety in relationships. These children are then more likely to develop internal working models characterized by expectations that others are unreliable and that they themselves are unworthy (Hawkins & Haskett, 2013). Chronic childhood interpersonal trauma therefore may come from child-caregiver relationships, but the internal working models that are formed as a consequence also impact how other relationships are formed later in life (Dugal et al., 2016). Indeed, studies have shown that children with experiences of developmental trauma show lower social functioning and have the conviction that others will not provide social support when asked (Messman-Moore & Coates, 2007). This may, in turn, hinder children with such convictions from utilizing the potential social support in their social network or from building that network to begin with.

In addition to children with PTSD not always being able to make use of their social network, the social network itself may also not be able to provide the social support a child requires. In cases of domestic violence, it is possible that the non-offending parent is also traumatized by the violence of their partner. Parents may experience PTSD symptoms themselves from a shared traumatic experience with the child or because of exposure to their child’s trauma (Tsang et al., 2021). Parental PTSD symptoms influence how parents speak to their children, being more likely to use a harsher tone (Murphy et al., 2016) and less likely to express positive emotions in conversations about traumatic experiences (Murphy et al., 2021) as compared to conversations about neutral topics. Beyond the direct impact of parental PTSD symptoms, children may also be impacted by a decreased ability of the non-offending parent to recognize the child’s needs and respond appropriately (McIntosh et al., 2021). A recent review has shown that mothers’ experiences of partner violence can trigger intergenerational trauma, with an impact on a child’s development and well-being due to the mother’s traumatic experience of domestic violence and related mental health problems (Graf & Schechter, 2024). Hence, parents may not be able to provide the support a child needs in the context of developmental trauma.

Besides a traumatized non-offending parent, it is also possible that the perpetrator of the trauma is part of the child’s social network, as is often the case in instances of interpersonal trauma (Shaw, 2010). Studies in adults show that those who have experienced betrayal trauma (i.e. trauma perpetrated by a close other; Kline & Palm Reed, 2021) are less likely to trust their romantic partner and others in general (Gobin & Freyd, 2014). In addition, people who experienced maltreatment as a child were also more likely to experience violence in their romantic relationships later, mediated by anxious attachment (Lee et al., 2013). This indicates that trauma perpetrated by someone from the social network may leave victims less able to make use of the social network as a source of social support due to trust and attachment issues. However, studies have also shown support from family and friends is particularly valuable in the case of childhood family violence (Sharp et al., 2017). While these latter studies are based on adult samples, it does show the importance of social support in cases of interpersonal violence. At the same time, the nature of the trauma may impact whether victims are able to make use of a supportive social network.

That is why the goal of the current study is to get a clearer picture of how children with interpersonal trauma perceive their social networks. As mentioned before, research on the use of social support and its impact in younger children is limited and has mainly taken a quantitative approach. An in-depth study of the perception of the social network and the support it provides could clarify how big and diverse the network is and which individuals are perceived as helpful in providing support. In this way, factors can be identified that could optimize the usage of the social network to positively impact PTSD symptoms in children.

To clarify the abovementioned gaps in the current knowledge, the current study aimed to answer two research questions: (1) How do traumatized children view the diversity and quality of peer relationships? and (2) How do traumatized children view the diversity and quality of family bonds? These questions were answered exploratively through in-depth semi-structured interviews with traumatized children aged 8–12. There was also room to discuss other individuals in the social network that were not defined as family or peers.

## Method

### Participants & Recruitment

The sample consisted of ten children between the ages of 8 to 12 (*M* = 10.00, *SD* = 1.63). Six boys and four girls participated. All children had suffered interpersonal trauma. Most children had witnessed or experienced parental abuse by their fathers (*n* = 9) and one child had been kidnapped at a young age and was later adopted. Eight children lived with their mother and two children with their foster/adoptive parents. On the Children’s Revised Impact of Event Scale (CRIES-13; Verlinden & Lindauer, 2017), a screening tool for PTSD symptoms among children, participants’ symptom severity scores on average indicated a high risk of PTSD diagnosis (*M* = 34.20; *SD* = 17.12).

The inclusion criteria were set up because the current study is part of a larger research project on social cognition and social network in children with PTSD. Children were eligible to participate if they were referred for trauma-focused treatment, between the ages of 8 and 12, spoke Dutch and had not previously been diagnosed with Autism Spectrum Disorder or a mild intellectual disability (because of known impairments in social cognition). All children that participated in this study were referred for trauma-focused treatment to the same trauma center at a child psychiatry clinic in the Netherlands. If children met the inclusion criteria, clinicians asked the guardians for permission to share their contact information with the researcher. Once guardians gave permission, the researcher would be notified and would call the guardians to explain the procedure of the study. Guardians that agreed to participate would then receive an e-mail containing the information letter and informed consent form. After consent was given, the interview would be scheduled.

### Procedure & Measures

Semi-structured interviews were conducted face-to-face at psychiatric clinic before trauma treatment started. Guardians were not present during the interview, but the child’s therapist was nearby and could be involved if children felt uncomfortable or overwhelmed at any point. When the child arrived for the interview, the researcher explained the procedure of the interview. Children were also reassured that they could stop the interview at any time if they felt uncomfortable. If the child agreed to the procedure, the interview started using an interview guide (Sloover et al., 2025). Children were first asked to define friendship, followed by more specific questions based on the interpersonal circles of closeness (Dietz et al., 2018). Here, every individual in their social network is placed within circles based on how close they feel they are to that person. Children started by writing their own name in the middle and then could write anyone else that they thought belonged to their social network within the circles, positioned based on closeness. For each individual, four specific questions were asked: (1) What do you like about (your relationship with) this person? (2) What do you not like about (your relationship with) this person? (3) Have you ever spoken to this person about the traumatic event that you experienced? (4) Has anything changed in your relationship with this person since the event? More in-depth follow-up questions were asked where fitting (e.g., when children mentioned they discuss their trauma with people, children were asked to describe how those conversations go and whether they were experienced as supportive). Children were encouraged to think about which family members and friends should be within the circles, but also other individuals that might be close to them (e.g., a neighbor or a teacher). All interviews were conducted one-on-one by the first author, who has a master’s degree in psychology and previous clinical experience in working with patients with PTSD. Interviews were audio recorded and lasted between 27 and 48 min (*M* = 35.9).

All legal guardians of the children gave written consent before the interview was conducted. Children provided verbal assent at the start of the interview. The Ethical Committee of Social Sciences at Radboud University (ECSW-2021-149) and the scientific research committee of the institution where data was collected (CWO2114) gave positive advice concerning the study procedures. The study was preregistered in the Open Science Framework (OSF; Sloover et al., 2023).

### Data-analysis

All interviews were transcribed verbatim by the first or second author. Then, a step-by-step reflexive thematic analysis (Braun & Clarke, 2022) was conducted, which is described in more detail below. Reflexivity was achieved through journaling. This study adopted a constructivist perspective, assuming that participants’ experiences are shaped through language and social context, and that themes are constructed through researcher interpretation. In addition to the thematic analysis, an overview was made of how many people were placed within each circle per child and whether these included family members, peers, or others.

#### Step 1: Familiarizing Yourself with the Data

The first step entailed the reading and re-reading of all transcribed interviews to identify some initial patterns. After the first six interviews were conducted, the first author began to read through the interviews and make notes of anything that seemed striking or important. Additions to the notes were made as more interviews became available.

#### Step 2: Generating Initial Codes

The next step was the coding of the transcriptions. Every piece of text that seemed relevant to the research questions was coded. New codes were made for data excerpts that needed to be coded but did not yet fit with existing codes. This was done transcript by transcript by the first author to ensure consistency in the coding. The second author checked all coded transcripts to make sure the codes were accurate and that no data excerpts were missed. Where deemed necessary, the second author suggested additional codes, which were adopted into the final coding. This step started when eight interviews were transcribed and was repeated for each new transcript that was added. All coding was done in ATLAS.ti (2024).

#### Step 3: Searching for Themes

In this step, the codes were first organized into themes. All team members looked at the codes individually and noted their first ideas on the themes. The team consisted of several expert members including a professor and assistant professor in developmental psychology and clinical psychologists who have experience working with the target group. Then the team came together for a full day to discuss all codes and themes and to come to an agreement on what the initial themes should be. This was done by going over each code one by one so there was agreement on what each code entailed. Next, all codes were written down on sticky notes to easily group and regroup them. All team members did this together through discussion. This step was done when nine interviews had been coded and transcribed.

#### Step 4: Revising Potential Themes

After the initial themes were agreed upon by the team, the first author revised the themes. An important consideration was whether themes accurately represent the patterns in the data (and codes). This was done when the final interview was also transcribed and coded to see whether any additional codes were created. While a few new codes were introduced, all codes from this interview fit within the existing themes. This was also checked by the second author.

#### Step 5: Defining and Naming Themes

In the next step, the first author named and described the themes. The names and descriptions were then checked by the second author. Agreement was reached through discussion. In addition, quotes were selected for every theme to give examples of what the themes reflect.

#### Step 6: Producing the Report

The final step entailed the writing process. An outline was written for the results section to decide in which order the themes should be discussed and how they were described. This outline was checked by one other team member (final author). After this, the full results section was written.

## Results

### Diversity and Quality of the Social Network

The results showed great variety in the number of individuals in the social network named by the children (min. = 7, max. = 23, *M* = 15.50, *SD* = 5.66). Most of these individuals included family members (*M* = 8.70) and friends (*M* = 5.80). These friends included classmates, peers at sports clubs and children from their neighborhood. In addition, some children also mentioned others (*M* = 1.00) who were not family members or friends, such as teachers, pets, friends of parents, and friends of siblings. On average, the inner circle contained more family members (*M* = 4.40) than friends (*M* = 1.40), while the second circle contained more friends (*M* = 2.30) than family members (*M* = 1.60). Interestingly, five children (50%) did place their father within a circle even though their father was the cause of their trauma. Three of these children placed him within the inner circle, while the other two placed him in the outermost circle.

Taking a closer look at the social networks, results showed that not all relationships were experienced as exclusively positive. On average, children reported in some ambivalent or negative way about 40% of the individuals in their network. Examples included mentioning bullying by friends or fighting family members. Out of the children who included their (perpetrator) father in their social network, two (40%) reported an ambivalent relationship with their father (i.e., there were good and bad sides to it) and three (60%) an exclusively negative relationship.

Regarding talking about the traumatic event itself, three (30%) children indicated never speaking to anyone in their network about it. Seven (70%) children mentioned having talked about the traumatic experience with someone in their network, of these children three did not experience these conversations as supportive. On average, children reported never speaking about their experiences with 85.62% of their network, having supportive conversations about the traumatic experience with 10.27% of their network and non-supportive conversations with 4.11% of their network.

### Themes

After thematic analysis, four themes were constructed: The parent-child relationship, defining friendship, defining support, and not discussing the traumatic experience. These are discussed in more detail below. The exact codes that were grouped with each theme can be found on the OSF project page (Sloover et al., 2025).

#### Parent-child Relationship

A recurring theme was the nature of the parent-child relationships, which seemed to be strained and have unclear boundaries. Children for example reported witnessing fights, hearing things that were not meant for them or being involved in fights between their parents. This is illustrated by the following quote:

*“One thing is less nice, he [dad] sometimes says that mom is holding dad back. Then mom says dad is dictating everything, then it’s also difficult for me if they are saying different things.”*

This example shows how parents can share their frustrations about the other parent directly with the child, which can put the child in a loyalty conflict. These unclear boundaries between parents and children could also result in role reversal, with the child taking a caregiver role as reported by some participants. Examples given were children excusing their parents’ behavior, addressing siblings’ behavior as if they were the parent, taking care of their parent or praising parents for doing household chores. One of the participants said:

*“My dad swears a lot and the other day my brother also said that. Now my brother* *says those things a lot and the other day we were at a playground and my brother said [swear word]… and then I said ‘now this has to be over*,* this is not fun anymore’. Yeah*,* my dad sometimes swears and my brother copies him”.*

This quote illustrates how a child can take over the role of a parent by telling their sibling that swearing is not allowed, even when the father is the one setting the (bad) example. As also shown in the examples above, the father-child relationship was often strained. Half the children did not name their father as part of their social network. The children that did place their father in the social network were not positive about this relationship. Among these children, some mentioned the presence of negative behaviors, such as their father swearing and treating them unfairly.

*“Well I often ask things and then it’s not allowed. I really wanted a Stitch teddy but that wasn’t allowed [by dad]. Then I went to stay with mom and she did buy me one and when I went back to dad he had bought my brother one. He didn’t give me one. The thing is that he gives the things that I want but he doesn’t allow, he does give to my brother”*

The quote above shows how a child experiences their father’s behavior as unfair, which is just one example of negative behavior child report about their fathers. One child mentioned seeing his dad more as a brother. Some children also showed some ambivalence in the relationship with their father:

*“He can be really sweet sometimes*,* but that is not always [the case]”.*

As opposed to their relationship with the father, children often reported a positive relationship with their mother. The majority of the children reported always going to their mother first for support when feeling sad and/or angry in general. However, only two children reported feeling supported when discussing the traumatic event with their mothers. Four children reported only discussing it because their mother brought it up, but that they did not feel supported in these conversations. So, while many parents (especially mothers) took up an important position in the social network of the child, this did not mean that these relationships were perceived as inherently supportive and could even be straining.

#### Defining Friendship

When asked to describe what friendship means, children often named positive qualities of a friend or things friends do, such as supporting each other, being nice, sticking up for the other, playing together, and not bullying each other. One child for example said:

*“A friend is when someone helps you, works together with you and does not bully”*

However, there seemed to be a contrast between this definition and their reality. When asked to describe their own friendships, children did mention positive qualities but also reported more ambivalent or negative factors. For example, children reported not knowing why they were friends with some of their peers, or what they like about that friend, or not knowing that friend very well. For example, when describing someone they named as a friend, a child said the following:

*“I’ve known him since second grade. Sometimes he’s nice and sometimes I don’t like him. Sometimes he’s very annoying. There really aren’t any good things about him, but bad things are also difficult to name.”*

When asked why the child then named him as a friend, he said:

*“I guess because he can be funny sometimes, but I don’t really know actually”*

The above example shows that children can show ambivalence in their friendship, with neither positive nor negative aspects and are not always sure of why that person would be a friend. In addition, negative behaviors by friends were also reported:

*“That’s when they bullied me the worst. She [friend 1] always wants to be the boss and then [name friend 2] does it too. Then I don’t understand anymore.”*

Other children also reported bullying by their friends as well as other negative behaviors, such as stealing friends. For example, when asked about potential negative aspects of a certain friendship, a child said:

*“I don’t like that she sometimes steals [friend] from me. By bullying, walking away and saying mean things”*

However, earlier in the same interview, this child had defined friendship as *“being helpful*,* being sweet*,* being nice to each other*”. So the behaviors that the peers she names as friends show, do not match the definition of friendship she gave earlier. It seems that children do know what friendship should entail, but it can be challenging for them to build positive relationships in their daily life.

#### Defining Support

When asked to describe the supportive individuals in their social network, children listed qualities such as being nice, helpful, and funny. Children reported on receiving practical support (e.g., help with homework) as well as emotional support. For example, one child reported emotional support from a friend when feeling sad:

*“He puts a hand on my shoulder and says it’ll be okay. He supports me”*

They also reported various approaches or responses that they experience as supportive regarding their traumatic experiences. Some children reported receiving tips on how to deal with the traumatic experience (e.g., writing down your feelings) or using humor to deal with the experience:

*“He [friend] talks about it with me and comes up with plans like rise up against the judge. And then we both start to laugh.”*

The abovementioned examples shows that there are various ways in which people in the child’s social network help the child cope with the traumatic experience. Another action that was experienced as supportive was discussing the traumatic experience with peers who can relate to the experience:

*“I can talk to him about my father very well. He [friend] has a mother who cannot take care of him and then it’s really nice that we are able to talk about that… It’s nice that we can talk about it without having to explain how the situation works.”*

Children seem to have varied ideas on what they experience as supportive from both parents as well as peers and what was experienced as supportive could be different for each child.

#### Not Discussing the Traumatic Experience

Even though there were a few things some of the participants experienced as supportive when talking about their traumatic experience, most children seemed to indicate that there were very few or no individuals in their network with whom they discuss their traumatic experience. Various reasons were reported for this, such as: does not feel the need to discuss it, it does not occur to them to discuss it, or not feeling comfortable with it. Some children also reported not getting the desired reaction when they previously tried to talk about their traumatic experience, such as others not understanding or not believing them:

*“Sometimes I say small things and then she [friend] says ‘right funny joke’ and I tell her it’s not a joke and continue with what I’m doing. And if then later I’m laughing again because I sort of want to hide how I feel, she says ‘see I knew you were joking’. So many children in my class can not really believe it.”*

This quote illustrates that peers may not always believe the traumatic experience to be true, which may lead to a child hiding how they feel and being less likely to try to discuss it again. Another child also gave an example of a negative reaction:

*“Because one time I wanted to tell him and then he laughed at me. And that hurt.”*

Such unwanted responses to an attempt at sharing reduces the likelihood of future sharing attempts. In addition, several children also reported not wanting to discuss their traumatic experience with certain individuals out of consideration for the other person’s feelings. For example, one child reported not discussing the traumatic experience with their grandmother because of her loyalty towards her son (child’s father).

*“I don’t really talk about it with her [grandmother] because she is very protective over my father and always chooses his side. Really loyal so to speak.”*

In addition, children reported that talking about the traumatic experience was hard for their mother, which makes it harder for their mother to support them:

*“My mom also struggles with it a little bit. So, she cannot help me as well as grandma or [step]dad. But she tries to help”*

Moreover, children also noticed their mother was struggling with the traumatic experience as their mother discussed it with others in front of them.

Another reason for not discussing the traumatic experience is the lack of a shared narrative as illustrated by the following quote:

*“One time we went through something traumatic and it is hard for her to deal with that. Then she makes the story differently in her head so that makes it sound like I am overreacting. So the difficult things that happened we can’t really discuss.”*

Hence, it is important for these children that they can share their experiences with a trusted other who can react in a sensitive manner, without the feeling they are burdening the other person.

## Discussion

The goal of the current study was to gain more clarity about how children with interpersonal trauma perceive social support within their social network. This allowed for identification of who and what in the social network may be experienced as supportive by children with PTSD. Results from semi-structured interviews with children who had experienced interpersonal trauma revealed that children generally had a diverse social network. Overall, children placed their family members closer to themselves than their friends. The social networks generally included very few individuals that children spoke to about their traumatic experience. Thematic content analysis revealed four themes: parent-child relationship, definition of friendship, definition of support, and not discussing the traumatic experience.

The results showed that the parent-child relationship was often strained. Some of the children who had experienced or witnessed domestic violence by their father, reported their father as part of their social network. However, children described these relationships as exclusively ambivalent or negative. Since the majority of these children had witnessed or experienced domestic violence by their father, it is reasonable to assume that their mother was also a victim in these situations. This points towards a challenge these children are experiencing: How do you shape a relationship with an abusive father when living with your mother, who was also a victim of the situation? Previous research has shown that a loyalty conflict towards abusive parents is a common phenomenon, specifically among foster children. Foster children often still view the bond with their birth parents as permanent and irrefutable, despite experiences of maltreatment (Dansey et al., 2018). Such convictions about maltreating parents can be explained by both attachment and betrayal trauma theory (Bernstein & Freyd, 2014), which states children often maintain relationships with abusive caregivers due to an evolutionary motivation to survive. The current study shows that this sense of loyalty can also pertain to abusive parents and poses significant challenges for the child.

At the same time, the relationship between child and (non offending) mother also seemed to be impacted. Previous research has already shown that difficulties in mother-child relationships can be long-lasting and even commence after separation from the abusive parent (Levendosky et al., 2002). This could be due to trauma symptoms the mother is experiencing. Parental PTSD symptoms have already shown to play a role in how mothers communicate with their children, often using a harsher tone (Murphy et al., 2016) and showing less sensitivity in trauma-related conversations towards their child’s needs (Meiser-Stedman et al., 2007). Another reason may lie in the lack of a shared narrative. Disagreements about the traumatic events that occurred appear to be a source of conflicts within families (Lindgaard et al., 2009). It is possible that children have different experiences than their mothers, which may make conversations about traumatic events uncomfortable or stressful. This could explain why few children in the current study experienced trauma conversations with their mother as supportive. Moreover, a lack of shared narrative also related negatively to overall family cohesion (Lindgaard et al., 2009), beyond conversations about the event(s) themselves. However, when mothers are experiencing trauma symptoms, this could form a barrier in discussing the traumatic events with their child and responding in a sensitive matter, thus preventing the construction of a shared narrative. Hence, relationships with both parents are impacted and could not always offer the support a child needs.

Aside from parental relationships, the current study also showed that peer relationships did not always match with how children would define friendship. A potential mechanism underlying these negative or ambivalent interactions with their friends could lie in a disturbed social cognition. Research in adults has shown that adults with PTSD have more difficulty recognizing other’s mental states (Sloover et al., 2022), are more likely to interpret other’s intentions as hostile (Ehlers & Clark, 2000) and are less able to recognize rules in social situations (Nazarov et al., 2014). While it is not yet clear whether these disturbances in social cognition also translate to children with PTSD, the internal working models of children with developmental trauma have been shown to be negatively impacted (Hawkins & Haskett, 2013), which shapes the way children interpret social situations. However, more research is needed to establish the role of internal working models in peer relationships of children with PTSD.

Generally, the results showed that most children had a relatively diverse social network composed of different family members and friends. The diversity of social networks plays an important protective role in adults with PTSD (Platt et al., 2014). However, most children reported speaking to very few or no one in their network about their traumatic experiences. As mentioned previously, this could be due to parental PTSD symptoms or child internal working models, but it could also be avoidance on the child’s part. Avoidance of thoughts and feelings associated with the traumatic events is one of the main symptoms of PTSD (American Psychiatric Association, 2013). Hence, children not talking to people about their experiences could be an expression of their PTSD, which is in line with children reporting “not wanting to talk about it” as a reason for not discussing it with others. Avoidance of such conversations by children also obstructs the chance of creating a shared narrative on the events with their mother. Besides avoidance as a PTSD symptom, it could also be that these children avoid to speak to their network about their trauma due to stigma within their social network. Victims often receive negative reactions from their network, for instance when still maintaining a relationship with the abuser (De Los Reyes, 2021). In addition, it could also be that these children have grown up in unsafe and harsh environments due to domestic violence, making it more likely they have developed an insecure attachment style (Bowlby, 1988). This not only impacts the parent-child relationship later in life, but is also predictive of more anxious and avoidant attachment towards other individuals later in life (Snyder et al., 2024). This forms yet another barrier for these children to make use of their social network for support.

The above mentioned results should be interpreted in light of a few limitations. Firstly, while the focus on interpersonal trauma is an important aspect of this study, it is uncertain whether these results generalize to different types of trauma. In cases of interpersonal trauma, the social network is already impacted due to the nature of these traumatic events (Shaw, 2010). While the current study emphasizes the impact on and importance of the social network in cases of domestic violence, it does not provide any information on the social network of children who were traumatized by different types of events.

In addition, the current study did not include a non-traumatized group of children. Although the results showed an important discrepancy between the definition of friendship and the way children describe their own friendships, it is not clear whether this same discrepancy would appear in non-traumatized children. It is possible that the ambivalent nature of some friendships is not something that pertains specifically to children who have experienced interpersonal trauma, but rather something that is generally the case in children without PTSD.

Despite these limitations, the results also lead to several suggestions for future research. As stated previously, internal working models and subsequent social cognition could play a role in the way children with PTSD build and benefit from their social network. So far, however, social cognition has only been studied in adults with PTSD. Future research should focus on the role of internal working models in children with PTSD and how it relates to their social network.

In addition, future research could take a similar approach as the current study but include children who experienced different types of traumatic events. Doing so would provide us with more knowledge on whether social networks are perceived differently by children who have experienced interpersonal trauma vs. children with non-interpersonal trauma. Another suggestion would be to add a control group with non-traumatized children to see whether the perceptions of the social network, specifically friendships, are different from the results of the current study.

In addition to suggestions for future research, the results have important implications for clinical practice. Most importantly, we should address trauma treatment in a systemic way. By ‘systemic’ we mean addressing trauma not just in the individual, but also in their relationships and environment. A child’s system is broad including both family members and peers. Parent-child trauma communication is difficult, for the child as well as the parent. Clinicians should be aware of this and offer support to parents to shape trauma related conversations. In addition, creating a shared narrative is important in parent-child trauma communication (Sloover et al., 2024). Clinicians could prepare the parent before having joint conversations about the traumatic experience and focus on improving the shared narrative of the events, which is beneficial to both parent and child (Lindgaard et al., 2009). Co-creating such shared narratives helps in understanding each others experience, but also acceptance of where their perspectives may differ. To achieve a shared narrative between mother and child, it is essential to go through a process where perspectives were shared and reactions to the experience were recognized (Lindgaard et al., 2009). This is for example done in Trauma-focused Cognitive Behavioral Therapy (TF-CBT). In TF-CBT, clinicians prepare parents and model sensitive reactions before having conjoint sessions with the child, where the child will share their trauma narrative (Diehle et al., 2015). Finally, clinicians should be aware of potential PTSD symptoms in parents. Parents could then be treated for their symptoms to improve parental sensitivity in trauma-focused conversations. That may in turn also improve the potential construction of a shared narrative.

In addition to parent-child interactions, it is important for clinicians to keep an eye on peer relationships. Merely asking if a child has friends is not enough, because having friends does not imply that these friendships can provide support. Therefore, it is important to ask about the quality of friendships to gauge whether children can actually benefit from any peer support and what children can do to improve this. In addition, support from peers who have experienced similar hardships may be beneficial. This provides children with an opportunity to speak to children who understand their experiences and experience peer support (Schechtman & Mor, 2010). The benefit of peer support groups has been widely recognized, for example in the context of bereavement by suicide (Krysinska et al., 2024). Children in such groups report that the main benefit for them was to meet other children who had similar experiences, as it helped them open up more easily and feel less alone (Metel & Barnes, 2011). While these studies were conducted in the context of suicide bereavement, it is plausible that this benefit would apply for children with interpersonal trauma as well, as the children in the current study report. It is thus worthwhile considering whether such support groups are available.

The current study revealed that children with PTSD still have a relatively diverse social network, but that they are not always able to benefit from this. This could be due to strained parent-child relationships and ambivalent friendships. Interpersonally traumatized children need embedding in their social network to benefit from support in their recovery, while the network is also affected by the trauma and its consequences. It is important for clinicians to be aware of this and find ways to support parents in trauma related conversations and children in strengthening their social network.

## Data Availability

Raw research data are not shared, as (guardians of) participants did not consent to this. However, the individual codes corresponding to the themes of the interviews are available on OSF (https://osf.io/ecbgw/). The preregistration and interview guide can be found here as well.
